# Acceptability, appropriateness, and feasibility of Rural School Support Strategies for behavioral interventions: a mixed methods evaluation over two years of a hybrid type 3 implementation-effectiveness trial

**DOI:** 10.1186/s43058-023-00478-4

**Published:** 2023-08-11

**Authors:** Hannah G. Calvert, Michaela McQuilkin, Ashley Havlicak, Teri Lewis, Lindsey Turner

**Affiliations:** 1 Center for School and Community Partnerships, College of Education, Boise State University, 1910 W University Drive, Boise, ID 83725-1742 USA; 2 School of Public and Population Health, College of Health Sciences, Boise State University, 1910 W University Dr, Boise, ID 83725-1742 USA

**Keywords:** School climate, Technical assistance, Virtual learning collaborative, Behavioral intervention, Implementation outcomes, Children’s health

## Abstract

**Background:**

Positive Behavioral Interventions and Supports (PBIS) is a framework for implementing evidence-based interventions for preventing behavioral issues and improving climate in schools. The implementation of school-wide PBIS with fidelity is complex, requiring leadership commitment, teaming, and coordination of systems for tracking behaviors and consequences. Putting these components in place while ensuring alignment with the values and needs of the school community can be difficult for schools with fewer resources, such as rural schools. Implementation supports are needed, including strategies such as technical assistance, but it is unclear whether lower-cost modalities such as virtual support are acceptable, appropriate, and feasible and whether perceptions vary throughout the implementation process.

**Methods:**

A type 3 hybrid implementation-effectiveness trial is taking place in 40 Idaho schools, testing a bundle of implementation supports selected to meet the needs of schools in rural areas. Supports include technical assistance from an implementation support practitioner (ISP), didactic trainings, virtual learning sessions, and an online resource portal. Surveys and interviews in the first 2 years of implementation (fall 2019 to spring 2021) explored outcomes of acceptability, appropriateness, and feasibility regarding the implementation supports among more than 150 school stakeholders.

**Results:**

Evaluations showed high acceptability and appropriateness of the PBIS concepts and training. The 20 schools receiving additional implementation support rated the technical assistance and support from the project’s ISPs as the most acceptable and appropriate resource. Reasons for acceptability were the relationship built with the ISP, the ISP’s expertise, and being a “neutral party.” Although in-person support from the ISP was preferred, remote support was acceptable and increased feasibility of attendance. Virtual learning sessions were acceptable for learning and collaboration, particularly in the second year of implementation, once ISPs had developed closer relationships with school teams.

**Conclusions:**

School staff found training, technical assistance, and virtual learning sessions to be acceptable and appropriate. Virtual formats of training and technical assistance decreased in acceptability but increased feasibility of attendance. In-person support was preferred during initial implementation, and virtual support was more acceptable thereafter.

**Trial registration:**

This trial was prospectively registered on ClinicalTrials.gov (NCT03736395), on November 9, 2018.

**Supplementary Information:**

The online version contains supplementary material available at 10.1186/s43058-023-00478-4.

Contributions to the literature

- We found that the use of both in-person and virtual modalities were acceptable and appropriate for delivering implementation supports, although acceptability varied based on timing in the implementation process
- Technical assistance from a trained implementation support practitioner was the most acceptable implementation strategy, compared to virtual learning sessions and an online resource portal
- Delivering training and technical assistance in the virtual format increased feasibility of attendance both for implementation support practitioners and training attendees
- Our findings contribute to the literature surrounding which supports are acceptable, appropriate and feasible for aiding scale-up of evidence-based interventions in rural schools

## Background

The Every Student Succeeds Act of 2015 mandated that US public schools use evidence-based practices not only to foster student academic achievement but also when providing mental health programming and other student services [1]. Although many universal interventions for improving student mental health and social-emotional learning have been shown effective in research [2–4], in practice, evidence-based programming that broadly supports students’ behavioral health and social-emotional needs is often not provided in schools or is provided with low fidelity [5–7]. Relative to schools in urban settings, at rural schools, the research-to-practice gap can be an even bigger challenge due to resource constraints, staff turnover, and inadequate funding [8, 9].

Positive Behavioral Interventions and Supports (PBIS) is a framework for implementing evidence-based interventions for preventing problem behavior and supporting student needs in schools [10]. While not a manualized intervention itself, PBIS is defined as a framework with a series of core features and best-practice guidance and tools to tailor those core features to fit a particular school context [11–13]. PBIS focuses on changing organizational systems and using school-based teaming, continued training, leadership involvement, coaching, and data-based decision making to effect change. PBIS is effective for improving student prosocial behavior and social-emotional functioning; reducing bullying, office discipline referrals, and suspensions [14–16] and improving school climate [17] and academic achievement [18]. The foundation of PBIS is the first tier—universal prevention—which involves schoolwide practices of establishing and teaching clear and consistent behavioral expectations and consequences for problem behavior, as well as rewards or acknowledgements for engaging in desired behaviors [13]. The adoption of PBIS is common in US schools but has been disproportionately higher among urban and suburban schools versus rural schools, with rural schools estimated to represent about 20% of schools implementing PBIS nationwide [19]. This does not reflect lesser need in rural areas, as youth in rural areas have less access to mental health resources [20] and two times higher rates of suicide [21, 22] compared to their urban peers. Identifying effective supports for rural schools is critical to scaling PBIS equitably, and studies of the implementation process are necessary to understand how best to facilitate effective scale-up in rural schools.

Implementation of evidence-based interventions (EBIs) in schools, particularly those with a universal focus such as school-wide PBIS, is dependent on a multitude of factors at the individual and organizational levels [8, 10, 18, 23, 24]. As noted in several publications about PBIS scale-up, literature and frameworks in implementation science have facilitated the understanding of PBIS implementation, particularly in defining the temporal nature of organizational change [25, 26]. Similarly, the vast knowledge base within the education sector regarding the application of multi-tiered systems of support, such as PBIS, is also a fertile ground for advancing the field of implementation science [27]. In particular, the use of multifaceted strategies to aid implementation of complex interventions is still a developing area [28, 29], as is the literature around scale-up of universal EBIs for promoting social-emotional wellness and mental health in schools [26, 27].

Various implementation strategies have been operationalized and identified as helpful for organizations (namely healthcare organizations) implementing EBIs through the Expert Recommendations for Change (ERIC) project [30–32]. The ERIC compilation’s strategies have also been translated for application in schools [33]. As described by Cook and colleagues, as well as in reviews of implementation supports including technical assistance (TA) [34] and active implementation support [35], there is much left to learn about how support strategies work and how they can be tailored and applied to various implementation scenarios. Part of what makes an implementation support strategy useful and translatable outside of research trials is its ability to be used by implementers (feasibility) and also how well it is received by implementers (appropriateness and acceptability). Components of acceptability, appropriateness, and feasibility were originally proposed as upstream implementation outcomes for EBIs themselves [31], but the same concepts can be applied to study the perceptions of implementation support strategies [36–38].

PBIS implementation practices dictate several components that are crucial to successful implementation, including the formation of implementation teams, high-quality training, the frequent use of data to make decisions, and ongoing action planning and feedback [11–13]. However, to support PBIS implementation with fidelity, additional supports are often needed [6, 26]. A recent systematic review that included 29 studies of the determinants of PBIS sustainment identified resources (including staffing, training, time, and funding) and ongoing training and support (including TA) among the top seven most important factors [39]. However, literature discussing how these supports should be tailored to rural schools, including which strategies are perceived as acceptable, appropriate, and feasible, is not well established. Rural school contexts, in particular, warrant special consideration due to features such as geographic isolation, limited resources, skepticism of outsiders, and poverty [40]. Importantly, identifying the features of implementation supports that make them acceptable, appropriate, and feasible for rural schools is essential for determining which strategies will be more likely to effect change and support scale-up.

Broadly, the selection and tailoring of implementation strategies to fit the needs of organizations in different contexts, including what strategies are most helpful *when* and *for whom* is an area of growing study [39, 41, 42]. Particularly, the literature discussing how implementation strategies should be tailored to rural schools, where proximity to implementation resources tends to be more limited, is not well established. This study examines perceptions of acceptability, appropriateness, and feasibility of a bundle of supports for facilitating PBIS implementation in rural schools. Specifically, we assessed the following: (1) perceptions of acceptability, appropriateness, and feasibility of in-person and virtual trainings among school implementation team members in 40 participating schools and (2) perceptions of acceptability, appropriateness, and feasibility of various in-person and remote enhanced implementation supports (including TA, monthly virtual learning sessions and asynchronous access to online educational materials and resources) among key implementers in 20 schools that had been randomized to receive these supports, in addition to training.

## Methods

The Rural K-12 project is a type 3 effectiveness-implementation hybrid trial testing Rural School Support Strategies (RS3), a bundle of supports for EBI implementation in rural schools.

RS3 could also be operationalized as a “blended strategy,” as it is a protocolized set of many discrete implementation strategies [31, 38]. Hereafter, we refer to general strategy categories (i.e., technical assistance) as “supports” while referring to the discrete activities within each support as “strategies,” where applicable. The results presented here reflect staff perceptions about supports and strategies during the first and second year of implementation (2019–2020 and 2020–2021).

### Theoretical framework

The larger project was conceptualized using the Quality Implementation Framework (QIF), which details the types of strategies that are best used at various stages of the implementation process [43]. The development of the QIF was partially informed by Fixsen and colleagues’ description of implementation stages [24], pertaining to PBIS [44]. The QIF details an exemplar implementation process and provides guidance to assist implementation, but it is not an evaluation framework [45]; thus, we applied Proctor and colleagues’ taxonomy of implementation outcomes as a guide for how to conceptualize the relevant features of the implementation supports and strategies [46].

### Study protocol

All rural public K-12 schools in Idaho with at least 100 students and no prior PBIS training were given the option to participate in the trial through mailed invitation letters and follow-up phone calls to the schools. Once 40 schools were recruited (three additional schools were waitlisted), they were randomized into two groups of 20 schools each: (a) training or (b) training and RS3 (described below). Implementation supports are further detailed in Table 1. Both training and the types of supports included in RS3 have been previously identified as important for schools implementing PBIS [41]. All schools received guidance in selecting a school-level coach—a staff member with key operational responsibility for leading PBIS implementation—and building a school PBIS team (i.e., 5–8 school staff members) to promote implementation. All teams attended a 4-day in-person training in summer 2019 and a 3-day virtual PBIS training in summer 2020.

**Table 1:** Timeline and modality of implementation supports used

	**Year 1 implementation**		**Year 2 implementation**	
**Implementation support description, including strategies used**	**Summer 2019**	**Fall 2019 to spring 2020**	**Summer 2020**	**Fall 2020 to spring 2021**
**Trainings** All schools completed the 3–4-day comprehensive tier 1 and tier 2 trainings. These included a mix of didactic lectures, full-group problem solving, and school team collaboration time. RS3 schools received three additional 1–2 day in-person trainings for school principals and coaches. These included a pre-implementation project kickoff meeting and coaching training in spring 2019 and another coaching training in spring 2020.	Training, in-person		Training, virtual	
**Technical assistance from ISP** RS3 schools received TA in the form of school visits or online meetings between the ISP and the school implementation teams and phone calls and emails between the ISP and team members. TA was flexible to the needs of the teams and included facilitation of team meetings, review of materials and/or data, help with action planning and problem solving, on-site observations and feedback, and other activities.	Email and phone call support	Monthly in-person school visits with ISP, email and phone call support	Email and phone call support	Monthly virtual meetings with ISP, email and phone call support
**Virtual learning sessions** RS3 schools attended these monthly webinars on various topics pertaining to implementation, given by the ISP and attended by the PBIS coach from each school. Coaches were also given time in breakout rooms to seek support from other school coaches. Topic examples included how to improve coaching skills and how to refresh implementation and focus on self-care in year 2.		Monthly virtual learning sessions		Monthly virtual learning sessions
**Online web portal** RS3 schools could access a web portal via personalized logins for each team member. The portal included downloadable resources from trainings, such as handouts and videos, as well as recordings of the virtual learning sessions. Users could also access a learning center with quizzes on content from trainings.		Web portal available	Web portal available	Web portal available

*PBIS* Positive behavior interventions and supports, *ISP* Implementation support practitioner, *TA* Technical assistance

### The Rural School Support Strategies (RS3)

The 20 schools randomized to the intervention condition (training + RS3) received supports throughout each year which included (1) in-person and remote TA on a proactive monthly basis from two experts on the project team (hereafter referred to as implementation support practitioners [ISPs], [35, 47]); (2) participation in monthly group-based virtual learning sessions with the ISPs, which included collaboration time with other school teams; (3) additional trainings to enhance coaching and leadership skills; and (4) access to a password-protected web portal with additional resources. The two full-time ISPs delivering the TA were both experienced K-12 educators with prior school leadership and coaching experience and had led PBIS implementation in Idaho schools. The monthly meetings between the ISP and each school were the primary delivery mode of TA. They were conducted on-site in September, October, and November 2019, then transitioned to virtual delivery (by teleconferencing) in December 2019 due to safety concerns with reaching remote rural areas in the winter (many required travel through snowy, mountainous regions). Originally, the design included conducting one onsite meeting at each school every spring and fall thereafter, but all meetings in 2020 and 2021 became virtual due to COVID-19 pandemic restrictions. Monthly meetings were centered around being responsive to schools’ needs and included elements of coaching, facilitation, problem-solving, and guidance on data-based decision making. See [48] for a full description of the trial methodology, including the strategies used, as classified by the SISTER Taxonomy [33]. See Additional file 1 for the completed Standards for Reporting Implementation Studies (StaRI) checklist [49].

### Measures

A concurrent triangulation mixed methods design was used to assess acceptability, appropriateness, and feasibility of the training and RS3 supports. Data were collected at many timepoints, including pre-implementation (tier 1 training surveys, summer 2019) and active implementation in the following order: the mid-year survey (December 2019), the year 1 interviews (spring 2020), ISP reflections (spring 2020), the tier 2 training surveys (summer 2020), and the year 2 interviews (spring 2021). Quantitative surveys provided information about attendees’ perceptions of the acceptability and appropriateness of the trainings, with additional write-in questions providing information pertaining to perceived feasibility (research question 1). For the training + RS3 schools, quantitative surveys were used to explore perceptions of the appropriateness of the TA and virtual learning sessions, with qualitative interview data allowing a more in-depth exploration of why each of the support components were or were not successful (research question 2). The qualitative and quantitative data from these various sources and different timepoints were given equal weighting, integrated during interpretation, and used in parallel to explore outcomes of acceptability, appropriateness, and feasibility of the RS3 supports [50]. The measures are further described below, and Table 2 details how specific items align with acceptability, appropriateness, and feasibility outcomes. Our interpretations for mapping the items onto constructs were informed by Proctor and colleagues’ descriptions of implementation outcomes [46] as well as Schultes and colleagues’ applications to the education context [51].

**Table 2:** Mapping of study measures onto implementation outcomes

	**Construct**		
	**Acceptability**	**Appropriateness**	**Feasibility**
**Quantitative data measures**	Training evaluations, year 1 and 2 survey items • The content increased my knowledge of the topic • The presenter was knowledgeable about the content • The presenter communicated information in a way that was easy to understand • The presenter was comfortable answering questions • The time allowed was enough to cover the content • I would recommend this event/activity to my colleagues • My overall rating of today’s session is favorable	Training evaluations, year 1 and 2 survey items • The content is applicable to my work • I need more explanation on the topics covered today • I need more support to implement this information Mid-year surveys, year 1 (training + RS3 only) • Survey items with the stem, “Were the following strategies helpful?” • Survey items with the stem, “Has the [support component] helped you do the following? Web site usage data, years 1 and 2 (training + RS3 only) • Number of individual users that logged in to web portal • Number of times each user logged in to web portal	Web site usage data, years 1 and 2 (training + RS3 only) • Number of individual users that logged in to web portal • Number of times each user logged in to web portal
**Qualitative data measures**	See below	See below	Interview prompts (training + RS3 only) • Year 1: What were the challenges to using [support component]? • Year 2: Did you have any barriers to attending the virtual learning sessions?
	**The following qualitative sources yielded themes that fell within various constructs:** Interview prompts (training + RS3 group only) • Year 1: How do you feel like the assistance from [the ISP] went? [follow-up prompt] did you have a preference for in-person vs. virtual assistance? • Year 1: How do you feel like the virtual learning sessions went? • Year 1: Were there any other supports that were particularly helpful? • Year 2: How has the virtual support from the ISP impacted your efforts to implement PBIS this year? • Year 2: How have the virtual learning sessions impacted your efforts to implement PBIS this year? Implementation support practitioner reflections, year 1 Training Evaluations, year 2 write-in response prompts • Compared to in-person training, what were the pros of completing an online training? • Compared to in-person training, what were the cons of completing an online training?		

Unless otherwise labeled, both groups completed the measures listed

#### Training evaluations

Members of each school’s PBIS team completed brief (17-item) evaluation surveys after attending the tier 1 training in summer 2019, and a second training in summer 2020, which reviewed previous content about schoolwide foundational practices, and expanded to include advanced (tier 2) practices. Questions were Likert-type or write-in response and assessed perceived acceptability and appropriateness of the training and feasibility of applying and intention/motivation to apply the content. This survey was designed by the practitioners leading the trainings, given a primary aim of the survey was to aid them in improving the quality of the trainings. Items used in the survey are commonly found in professional development evaluations in the K-12 education domain [52].

#### Mid-year surveys

In December 2019, school PBIS coaches at training + RS3 schools completed an online survey assessing the appropriateness of training and supports they had experienced so far. This survey was designed by the research team.

#### Interviews

The school PBIS coach and principal from training + RS3 schools were each interviewed (separately) at the end of year 1 (April/May 2020). In year 2, only the school coach was interviewed (February/March 2021). Interviews lasted approximately 30 min, using a semi-structured guide. Trained interviewers (HC, MM) conducted interviews via Zoom and recorded and transcribed verbatim for analysis.

#### Implementation support practitioner reflections

The two ISPs provided written reflections using structured prompts in an open-response format. They addressed the benefits and challenges of in-person and virtual TA.

### Data analysis

#### Quantitative

Demographic data for the schools, including rural locale and its subtypes [53], were obtained from the National Center for Education Statistics and summarized using descriptive statistics. Descriptive statistics were calculated for the summer training evaluations and mid-year surveys. Independent sample *t*-tests were used to compare year 1 and 2 training evaluations. Individual-level changes over time for training surveys were not evaluated, as the surveys were anonymous. Usage of the online resource portal was tracked by user (all PBIS team members at training + RS3 schools had individual login access) and date using Google Analytics and analyzed descriptively. Attendance to the trainings is not reported as a feasibility outcome because the research team set limits on how many people could attend each year. An adjusted critical *P* value of < 0.001 was used to account for multiple comparisons. Analyses were computed using SPSS version 29 (IBM SPSS Statistics, Armonk, NY, USA).

#### Qualitative

De-identified interview transcripts were coded and analyzed using Dedoose Version 8.3.45 (SocioCultural Research Consultants, LLC, 2016). Interview analyses were done in two cycles [50, 54]. Transcripts were first divided into excerpts by question, then open coding was done by a single coder (MM) in the first cycle to conceptually code response excerpts based on the question that was asked. Both coders (MM and HC) met several times to discuss the concept coding strategy, and reached agreement on the coding style for 10% of transcripts before completing the rest of the coding independently. Thereafter, the two coders independently reviewed all excerpts and conducted a preliminary thematic analysis [55]. Coders met three times to discuss and modify emergent themes and finalize theme descriptions. Write-in responses on survey items were open-coded by a single coder (HC) using content analysis [56].

## Results

Results are presented below by support component and data source/year, where appropriate. Regarding the interview data, interviews with school coaches and administrators at training + RS3 schools solicited perceptions about all RS3 components. During the second year of the project (2020–2021 school year), COVID-19 was continuing to impact schools. Year 2 interviews with training + RS3 school coaches were shortened to reduce burden and only asked about the most frequently utilized elements of RS3: TA from the ISPs and the virtual learning sessions. Demographic characteristics of schools are provided in Table 3. More exemplar quotes are presented in Additional file 2.

**Table 3:** Demographic characteristics of 40 participating schools, by condition

	**Schools randomized to training + RS3 (*n* = 20)**			**Schools randomized to the training only (*n* = 20)**		
	**Mean (SD)**	**Min**	**Max**	**Mean (SD)**	**Min**	**Max**
Number of students at each school	334.2 (184.9)	94	681	363.4 (173.2)	161	780
Number of classroom teachers at each school	17.9 (7.9)	6	36	19.6 (6.1)	12	32
Student-teacher ratio	17.9 (4.0)	10.3	26.2	17.9 (3.9)	9.9	25.3
Percentage of students at school eligible for free/reduced-priced meals	46.0 (19.0)	17.1	91.8	51.0 (16.7)	28.3	92.6
	**Number of schools**	**%**		**Number of schools**	**%**	
Percentage of students at each school eligible for free/reduced-priced meals						
< 40% students eligible	8	40%		6	30%	
40–60% students eligible	8	40%		9	45%	
> 60% students eligible	4	20%		5	25%	
Remoteness (all schools within rural/township locale)						
Fringe	3	15%		4	20%	
Distant	8	40%		9	45%	
Remote	9	45%		7	35%	
School level based on grades served						
Elementary only (grade 6 or lower)	12	60%		11	55%	
Elementary/middle (K to grade 8)	1	5%		0	0%	
Middle school (grade 6 to grade 8)	2	10%		4	20%	
High school only (grade 9 to grade 12)	2	10%		2	10%	
Middle/high (grade 7 to grade 12)	1	5%		1	5%	
All grades (K to grade 12)	2	10%		2	10%	
Total number of students across all schools	6684			7268		
Total number of teachers across all schools	357			392		

Data source is the 2018–2019 Common Core of Data, National Center for Education Statistics. *RS3*, Rural School Support Strategies

### Trainings

#### Training evaluation survey data

Descriptive statistics from all school staff responses on the training evaluation surveys are listed in Table 4. There were no meaningful differences between staff from training and training + RS3 schools. In both years, ratings on items measuring acceptability and appropriateness were high (> 4/5). However, independent samples comparisons showed lower ratings for several items in year 2 compared to year 1. Perceived applicability of the content, the novelty of the content, the clarity of presentation of the content, the motivation and plan to use training concepts in their work, positive recommendation of the training, and overall training quality were all rated significantly lower in year 2 compared to year 1. The magnitudes of these changes were small, with 0.44 as the largest decrease. The two items that significantly improved for year 2 were “I need more explanation” and “I need more support,” with a 0.69 and 0.65 decrease, respectively.

**Table 4:** Perceptions of PBIS trainings, by year (all schools)

	**Year 1 in-person training, July 2019**	**Year 2 virtual training, July 2020**
	*n* = 163 *n* = 93 training only *n* = 70 training + RS3	*n* = 154 *n* = 75 training only *n* = 79 training + RS3
	**Mean (SD)**	**Mean (SD)**
The content is applicable to my work	4.88 (0.43)	4.54 (0.77)*
The content increased my knowledge of the topic	4.81 (0.48)	4.53 (0.73)*
The presenter was knowledgeable about the content	4.87 (0.44)	4.67 (0.83)
The presenter communicated information in a way that was easy to understand	4.82 (0.47)	4.38 (0.89)*
The presenter was comfortable answering questions	4.83 (0.46)	4.61 (0.86)
The time allowed was enough to cover the content	4.12 (1.0)	4.12 (0.91)
I am motivated to use what I learned today	4.80 (0.48)	4.47 (0.75)*
I would recommend this event/activity to my colleagues	4.77 (0.52)	4.40 (0.81)*
I plan to use this information in my work	4.90 (0.41)	4.56 (0.70)*
I need more explanation on the topics covered today	3.31 (1.1)	2.62 (0.98)*
I need more support to implement this information	3.51 (1.1)	2.86 (0.91)*
My overall rating of today’s session is favorable	4.78 (0.50)	4.53 (0.74)*

Response options range from 1 = strongly disagree to 5 = strongly agree: *p < .001 for Y1 to Y2 comparisons, within group

School teams attending the year 2 virtual training were also asked to write comments about pros and cons of the online modality, in an open-response format. Commonly stated pros were about flexibility/convenience and team collaboration time. Attendees did not have travel hassles or time away from family, which made attending the training feasible for more team members. Many stated that the flexibility of being able to talk more with their teams and walk around the room without disturbing other groups was valued. More-effective team collaboration time (increased efficiency, more conversations, fewer distractions/noise from other groups) was a benefit.

Reported drawbacks of virtual training were varied. Attendees missed being in the room with instructors, as well as other school teams, describing that the comradery and collaborative learning possible in person was not possible to replicate in the virtual format. The lack of personal connection came up regularly. Many noted that it was difficult to stay engaged online, whereas the physical proximity of the instructors at in-person training helped teams stay on task and get immediate help. The online format made asking questions more difficult, despite the multiple avenues provided (chat box, breakout sessions, open forums after lectures). Staff noted that having their teams not in the same room (some teams attended separately for COVID-19 physical distancing reasons) made teamwork harder. Teams that attended while in the same room (i.e., each team in a conference room at their school building) noted improved collaboration. Technical difficulties were infrequently noted.

Taken together, survey items and open responses showed that the training was highly acceptable and appropriate both years, but with slightly lower ratings on presenter communication, content applicability/novelty, motivation/plan to use the content, and overall rating of the training in year 2. Qualitative findings elucidate that decreased acceptability and appropriateness of the year 2 training likely stemmed in part from the virtual format to the in-person training format. However, feasibility of attending the training increased.

#### Mid-year 1 coach surveys

Results of the mid-year (December 2019) training + RS3 school coach surveys are listed in Table 5. At least half of the coaches surveyed reported “very much” regarding the project kickoff meeting and the coaching institute being helpful for implementation. This rating was higher than the virtual learning sessions, but lower than the TA from the ISP.

**Table 5:** School coach perceptions of appropriateness, RS3 + training schools only (*n* = 20)

Items	**Not at all**	**A little**	**Moderately**	**Very much**
**Were the following strategies helpful?**				
State Conference in 2019		2 (11%)	8 (42%)	9 (47%)
Project Kickoff Meeting		1 (5%)	9 (45%)	10 (50%)
Coaching Institute		1 (5%)	6 (30%)	13 (65%)
Summer visit from ISP			5 (25%)	15 (75%)
Fall 2019 in-person visit(s) from ISP			4 (20%)	16 (80%)
Virtual learning sessions		4 (20%)	8 (40%)	8 (40%)
**Has the technical assistance [meetings with ISP] helped you do the following?**				
Identify resources		2 (11%)	4 (21%)	13 (68%)
Increase knowledge			3 (16%)	16 (84%)
Improve decision-making			3 (16%)	16 (84%)
Use data for decision-making		1 (5%)	5 (26%)	13 (68%)
Resolve challenges			4 (22%)	15 (79%)
**Have the virtual learning sessions helped you do the following?**				
Identify resources	1 (6%)	1 (6%)	7 (39%)	9 (50%)
Increase knowledge		1 (6%)	5 (28%)	12 (67%)
Improve decision-making	1 (6%)		7 (39%)	10 (56%)
Use data for decision-making	1 (6%)	2 (11%)	5 (28%)	10 (56%)
Resolve challenges	1 (6%)	1 (6%)	7 (39%)	9 (50%)

Sample sizes ranged from 18 to 20 due to item-specific missingness, *ISP* Implementation Support Practitioner, *RS3* Rural School Support Strategies. Data are from December 2019

#### Year 1 administrator and coach interview themes

When coaches or administrators at training + RS3 schools were provided an open-response interview question about support components that they found helpful aside from the TA and virtual learning sessions, they most often cited the training, which all schools received. Themes are described as follows.

##### Team collaboration time

During the trainings, PBIS teams were able to plan for the upcoming school year. One principal articulated the power of the training sessions:

The big thing, honestly, was the training… We got the time that we needed to really sit down and be intentional about, how are we going to roll this out, what are some of the oppositions we're going to face and what are we going to do about it?

##### Quality

Participants said that there was a lot of high-quality content at the training, but the amount of information made it difficult to retain. A principal conveyed this, saying “When we were in summer conference, there was so much information... Everybody in my team was taking notes like crazy, but there are just some things that we forgot.”

### Technical assistance from the ISP

#### Mid-year 1 coach surveys

The highest-rated support component on the survey was in-person TA visits from the ISP (80% rated “very helpful”; Table 5). More than two thirds of the coaches surveyed reported “very much” regarding the virtual learning sessions helping them to identify resources, increase knowledge, improve decision-making, use data for decision-making, and resolve challenges.

#### Year 1 administrator and coach interview themes

Participants were overwhelmingly positive about interactions with their ISP, indicating high acceptability and appropriateness of the support. Themes are illustrated below.

##### Knowledge

Participants said the advice their ISP gave was always helpful and their expertise was valued. Having an expert who had first-hand experience working in schools and with PBIS implementation was viewed as a positive. One coach noted, “His insight and knowledge on PBIS really helped to pave the way and kind of give us our next steps when we felt like, where do we go from here?”

##### Presence

While some school teams used the help of the ISP a lot, and some less, all reported that having the ISP as a resource was helpful. A coach stated, “He was right there ready to help us out in any way that we needed… ask him any questions, he could answer them.” Participants also said that the knowledge that the ISP would attend (physically or virtually) their school PBIS team’s meetings spurred them to get their agendas and data in order, and be ready with questions. A principal stated, “Accountability is what makes stuff work when you know you have to…you’re going to have to tell him what you’re doing.”

##### Relationship

Starting with the year 1 summer training and continuing throughout the school year, each ISP was intentional about building relationships with coaches and teams at each school. This emerged in interviews as participants expressed genuine appreciation and respect for their ISP. These relationships were grounded in the approachable and open communication style of the ISP and in connecting over similar experiences. The connections helped built trust between the ISP and the rest of the school staff. Sharing personal connections helped to foster relationships even more quickly, as stated by a coach, “We hit it off with [the ISP] because he is from [here] originally, so he knows the dynamics of what we're working with.”

The position of the ISP as someone outside of the school gave their opinions additional weight. One coach noted, “Having the meetings with [the ISP] was super helpful [for] mentorship and guiding, and a third party looking above and seeing what he sees objectively.”

##### Communication style

Many coaches and administrators stated that they really valued the professional and positive way that their ISP interacted with their school PBIS team. The ISP was valued for their willingness to listen without judgment, providing advice only when needed. The PBIS teams led the meetings, with the ISP stepping in to guide and offer suggestions, rather than telling teams the “correct” way to do things. One principal explained, “He allowed us to really vet ideas without dominating the conversation… he was really supportive, but also approachable.” Participants noted that constructive comments were always delivered with positivity, modeling how the ISP was encouraging the team to interact with their colleagues and students. A school coach talked about communicating with the ISP, “I've appreciated him reaching out to me and saying, ‘You're doing a great job.’ I've just always felt supported by it." Another coach said, “Even when there are things that we need to improve on, it's always positive.” Participants appreciated that the ISP reinforced the things going well, rather than focusing only on areas that needed improvement.

##### Delivery method

Face-to-face interaction was preferred over virtual meetings or interacting over email or phone. Drawbacks of virtual meetings included technical issues, decreased engagement, and decreased ability to read body language. In-person visits allowed for better relationship-building. Many stated that they would bring the ISP on a building walk-through to meet teachers and observe classroom behavior, and to provide suggestions. One coach noted, “Our staff was much more… willing to stick ideas out there and bounce them off [him] when he was in the room versus when he was on the screen.” In-person visits also allowed staff the opportunity to ask the ISP sensitive questions privately. Most stated that virtual support was still appropriate (and better than not having any TA). The initial mode being in-person eased the transition to virtual meetings: “He had established relationships with us enough before he went to the online, that [the transition] wasn't major. I do think that we got more [in person]. Face to face is more powerful.”

#### Year 2 coach interview themes

In year 2, TA from the ISP was still seen as highly acceptable and appropriate; two themes recurred, and a third theme emerged:

##### Presence

Coaches were still happy to have the ISPs available and emphasized that the ISPs always responded very quickly when they reached out for help. Even when coaches did not reach out, the fact that the ISPs proactively contacted teams regularly helped to keep PBIS going during an unprecedented year: “It's been a hard year, it's been hard to prioritize everything and they keep reminding me 'Okay, yes, PBIS is still a priority for us, we've got to keep going'. So that has helped a lot.”

##### Relationship

The relationship built between the school coach and the ISPs was invaluable. There was genuine care for their wellbeing that made them feel even more supported; one coach said, “I really feel like he cared about me as a person and not just about how PBIS was going at our school.” There was an overwhelmingly positive response regarding their involvement.

##### Flexibility

In year 2, the ISPs focused on supporting teams in whatever capacity they needed, and the support—without pressure to do more than teams could handle—was appreciated. ISPs helped teams set realistic goals and assuaged worries about regressing in some areas of implementation. One coach said, “They've kind of pulled back which I think is probably where they need to be this year. They let us know they're there if we need them.”

#### Implementation support practitioner reflections

Each ISP independently documented perceptions about the in-person and virtual formats for delivering TA. They expressed that in-person visits were highly acceptable and appropriate. Benefits included better relationship-building with school staff, ability to facilitate deeper conversations and better address specific challenges/sensitive issues, and increased familiarity with the physical school site and school climate. Visiting sites helped the ISPs develop a richer understanding of the strengths and needs of each school, be more involved with establishing processes, and prompt school teams to keep meeting. The benefits of virtual support included higher feasibility of attendance for the ISPs (more flexible scheduling, less time traveling). It was appropriate for quick updates after rapport was established with teams from the in-person visits, for screen-sharing documents, and there were no travel/health risks. However, technical challenges hampered productivity, and it was harder to have private conversations and to model processes virtually. Overall, the opinions of the ISPs on the acceptability, appropriateness, and feasibility of the in-person versus virtual TA corroborated the statements of the school coaches and administrators.

### Virtual learning sessions

#### Mid-year 1 coach surveys

At least half of the coaches surveyed reported “very much” regarding the virtual learning sessions helping them to identify resources, increase knowledge, improve decision-making, use data for decision-making, and resolve challenges (Table 5). This represented overall lower appropriateness for the virtual learning sessions compared to the TA from the ISP.

#### Year 1 administrator and coach interview themes

Participants had mixed feelings about the acceptability of the virtual learning sessions, although responses were generally positive. Themes are described below.

##### Review

Many reported the virtual learning sessions as useful for reiterating concepts presented previously, since the large volume of new information in the multi-day training sessions could make it difficult to remember certain components.

##### Collaboration

Coaches expressed that it was helpful to troubleshoot implementation problems with coaches from other schools. One noted, “At first I kind of thought ‘this is just one more thing and isn't going to be helpful.’ But it's been nice to have an opportunity to talk to other schools, and to [compare] our progress [to] where they're at and see their challenges and help each other.”

##### Delivery method

Some stated that the digital interface of the virtual learning sessions was a drawback, either because teleconferencing made it harder to block out distractions or it was not conducive to their learning preferences. One coach stated, “If you're there in person, you're held a lot more accountable than if you're online.” Technical issues, such as internet connectivity problems and challenges with Zoom, did come up for some.

#### Year 2 coach interview themes

The virtual learning sessions in year 2 had a larger focus on stress management and self-care at the start and shifted toward PBIS later in the year. The collaboration element of the sessions was more acceptable and appropriate in year 2.

##### Review

Participants noted that the self-care focus of the virtual learning sessions was appreciated because there was so much stress and uncertainty. Staff appreciated the reminder to take care of themselves.

##### Collaboration

The value of relationships that teams had built with other schools in the project was highlighted in year 2: “I feel like I actually know [the other coaches] now. Because we're always in breakout sessions… and we do work, but it's nice to catch up.” Participants became better friends, and breakout rooms during the virtual learning sessions helped them solve problems and not feel as isolated. Another coach said, “I really like being able to talk to people at different schools to see if they're on the same page as us …it's just nice to see how everyone's doing …we're there for each other.”

##### Delivery method

While some staff noted the ease of participating such as, “I can listen on my phone,” others had consistent conflicts with other school events, and some would not participate at all because it was virtual. Technical barriers were less of an issue in year 2.

### Online resource portal

#### Online resource portal usage data

During the 2020 calendar year, 9 users from 6 of the 20 training + RS3 schools logged in and accessed various resources within the portal 64 different times. As measured by access dates, 70% of the portal usage occurred prior to the COVID-19 school closures.

#### Year 1 administrator and coach interview themes

The web portal was cited as an appropriate resource repository, when participants thought to use it. The single theme is described below.

##### Review

Participants used the portal to find video content from trainings, recorded webinars, and documents (e.g., blueprints, planning forms). A coach said, “I watched some of the video tutorials to help refresh my memory.” Few reported accessing the portal independently, rather they did so after a referral from the ISP regarding a specific resource.

## Discussion

This study assessed perceptions of over 150 rural school staff members about supports for implementation of school-wide PBIS across several years. The findings shed new light on when and why certain implementation supports, including training and TA in in-person and virtual formats, are considered acceptable, appropriate, and feasible for implementers in rural schools.

Results from all schools showed high ratings of perceived acceptability and appropriateness of the content and quality of the in-person training in year 1. Interviews with school coaches and administrators reiterated that the training was valuable, and dedicated collaboration time for school teams was important. Combined results from the surveys and interviews illuminated that the trainings covered an extensive amount of material which was hard to digest in the time allotted, which has been noted about PBIS trainings previously [57]. For teams at training + RS3 schools, having other opportunities to review and re-learn this material during the virtual learning sessions throughout the year, and to review it during dedicated meetings with their ISP, was helpful.

In year 2, training was delivered virtually. School stakeholders still rated the training as high quality, but average responses were significantly less favorable in several dimensions (e.g., acceptability, appropriateness, intent to use the information learned). While it was not directly assessed through the surveys, it was clear from the interviews that the ongoing COVID-19 pandemic affected perceptions of the relative priority and feasibility of implementing PBIS. In general, readiness is an important factor for implementation of universal EBIs in schools [58], so implementation stage (due to the pandemic, or other factors) may have also affected participants’ perceptions of the training and intent to apply the concepts. When participants specifically reflected on the transition to the year 2 virtual format in an open-response, most expressed that in-person training was more acceptable and engaging. The online format was missing the human element of connection with the instructors, the ease of asking questions, and the collaboration that comes from being in the room with other school teams. However, there were benefits of virtual trainings—namely increased feasibility of attendance and effective team collaboration time—indicating that the virtual format is appropriate but perhaps more suitable for ongoing activities such as the virtual learning sessions.

Other published data from PBIS trainings during the pandemic corroborates that in-person trainings can be more acceptable due to fewer distractions that arise from staff members’ duties in the school building, as well as better access to the instructors. Data from same survey found that team collaboration time is the most highly rated element of virtual trainings, and that appropriateness of trainings (including the clarity of the concepts) and feasibility (using the knowledge gained) was lower for schools implementing PBIS during the COVID-19 pandemic [57]. Additional benefits of virtual trainings cited in the literature include increased accessibility for rural communities as well as greater potential to tailor trainings to various needs within school teams (e.g., new team members who are catching up, or differentiated training content based on team role) [59].

When coaches at training + RS3 schools rated the acceptability of the RS3 supports, it was clear that the monthly meetings with the ISPs were the most valued, followed by the in-person trainings, the monthly virtual learning sessions, then the online resource portal. No strategies were rated as “not at all helpful,” so it could be argued that having more resources is better; however, it is important to consider the material and labor costs behind less-favorably rated components such as the online portal when considering how to deliver support. ISP meetings were seen as more appropriate for helping school teams with tasks such as data-based decision making and resolving problems. This corroborates other studies showing that TA can facilitate these aspects of PBIS implementation [60].

Our analysis of interview data revealed aspects of the RS3 components that increased perceived acceptability and appropriateness. As shown in prior work, the relationship between the support specialist and the school team was important for acceptability [34, 61–63]. In the early implementation phase, when teams and ISPs were just starting to build their relationship, the fact that the ISPs had previously worked in rural schools in Idaho helped create more immediate trust and rapport. In Year 2 with the stress of COVID-19 ongoing, the relationship between coaches and ISPs was a primary reason school coaches felt it was possible to continue PBIS implementation.

The delivery modality of the TA was also important; in-person visits were essential early in the process to build rapport. Once relationships were better established, virtual meetings were more acceptable and appropriate. As noted in a recent systematic review of the mechanisms through which ISPs are able to improve implementation outcomes, the characteristics and competencies of ISPs are crucial for success [35]; the personal characteristics, behaviors, and micro-communication skills of the two ISPs on our project appear to have been highly effective for building trusting relationships with school implementation leaders. Elements that appeared to accelerate relationship-building included the background of the ISPs working in schools similar to those implementing in the study, as well as their willingness to “meet schools where they were at,” or provide the help that was needed without judgment. Factors identified as important for trust building in the ISP-implementation team relationship in other recent work [35, 47] were described by coaches and administrators: communication style (i.e., empathy-driven exchanges, authenticity), presence (i.e., predictable and frequent interactions), and knowledge/demonstrated expertise were all identified elements which increased the acceptability of the TA in our study. Additional research exploring the interpersonal elements of the support process seems well-warranted given how central it is to organizational stakeholders’ perceptions of acceptability and their engagement with implementation support interventions.

In year 2 when all TA was virtual, schools reported few logistical drawbacks to the virtual modality but still expressed that they missed the in-person connection. Other research has shown that delivering school mental health-focused virtual training and TA can be effective, acceptable, and, importantly, can provide more-equitable access for those not typically able to afford the time or costs of travel [64, 65]. ISPs reflected that the virtual format made it more feasible for them to attend school-level meetings.

The virtual learning sessions were well-regarded in both years as a tool to review material from the trainings. Some coaches disliked the virtual format, but many found it worthwhile, particularly the breakout sessions where coaches from different schools could problem-solve together. By year 2, school staff had formed closer relationships and were leaning on one another for information and emotional support. The rapport built between school coaches as the study went on appeared to increase the acceptability of the virtual learning sessions in the second year compared to the first year, showing that virtual community-building with staff from similar school environments is a helpful implementation strategy. This type of collaboration can benefit staff located in geographically remote areas (e.g., where there is a single elementary school in a district), where opportunities to collaborate with peers from other schools are scarce [66]. This finding contributes to the growing literature on the use of online communities to aid in professional development and implementation of EBIs [67, 68]

Strengths of our work include the longitudinal data collection, breadth of stakeholder opinions assessed, and use of mixed methods to explore opinions in depth. Limitations include the use of quantitative data collection measures that were specific to this study. Though psychometrically strong measures for examining acceptability, appropriateness, and feasibility exist [69, 70], our measures were also used to inform the ISPs on how to improve support components in real-time and thus were tailored to those needs. COVID-19 introduced unexpected challenges, including that training and ongoing support were required to be virtual for all schools starting in March 2020; opinions of virtual/remote strategies may have been affected by the lack of other options. We acknowledge that the implementation strategies we report in this paper are underspecified [28] and that our assessments of acceptability, accessibility, and feasibility were not parsed out into specific strategies as articulated in the SISTER taxonomy (i.e., coaching, tailored feedback). Opinions of stakeholders at schools where the leadership has chosen to adopt PBIS, such as those in this study, may be inherently different from those at schools that have not. While this paper does not explore the relationships between implementation supports and subsequent fidelity of implementation, those data will be reported in forthcoming work.

Implications for trainings and technical assistance To maximize acceptability and feasibility, district or state systems seeking to provide support for scale-up of EBIs such as PBIS in rural schools should consider the following practices: if it is not cost-prohibitive, provide in-person training when delivering multi-day, multi-group sessions with a lot of content. Follow-up refresher trainings or shorter trainings are more acceptable in an online format. To increase access and feasibility for all participants to attend “in-person,” offer online content (such as previously recorded lectures, or livestreaming the training) for viewing locally and an opportunity for teams to have collaboration time in hybrid format if desired (i.e., help teams navigate having remote participants videoconference for collaboration times).

For virtual trainings, having well-rehearsed, quality content, and appropriate scheduling for teams to have dedicated work and break time is critical. Furthermore, having several experts on hand (who are not actively presenting) to answer real-time questions in the chat, and provide a breadth of coverage to attend to questions during groups’ collaboration time in breakout rooms, will increase perceived acceptability of the virtual format. Providing online access to lectures for participants to watch before or after the trainings (whether in-person or virtual) increases participants’ ability to process the content.

Regarding implementation support/TA, it must be provided by specialists who are experts not only in knowledge of the EBI but are also highly competent in relationship-building and positive coaching skills. Site visits from specialists are highly appropriate and acceptable during adoption/initial implementation. Appropriateness could vary based on the scale of the intervention; for systems-level changes/complex behavioral interventions such as school-wide PBIS, in-person visits foster relationship-building, increase stakeholder buy-in, and immersion in the school context aids problem-solving. Offering a mix of in-person and remote support after initial implementation, depending on feasibility (budgeting, time constraints), and needs of stakeholders, is appropriate. Regarding online resource portals, they can be viewed as an acceptable and appropriate resource but may not receive much use if there are other person-centered forms of TA that do not require use of the portal. If resources can simply be sent as an attachment or link in a targeted TA email (such as to YouTube, etc.), developing a repository may not be the best use of resources, so cost to the provider is an important consideration.

## Conclusions

We found that in-person and virtual trainings with ample collaboration time for teams, and ongoing implementation support provided in-person or virtually by an experienced ISP, were highly acceptable and appropriate strategies for supporting PBIS implementation in rural schools. Other supports, including monthly virtual learning sessions and a web portal with resources, were acceptable, but not as highly rated. While school staff prefer in-person trainings and meetings with ISPs when possible, strategic use of virtual meetings and TA (such as in-person at the onset of implementation, to better build relationships—and virtual later on) increases the feasibility of providing high-quality support to schools in remote settings, while not compromising too much on acceptability and appropriateness.

### Supplementary Information

**Additional file 1.** Standards for Reporting Implementation Studies: the StaRI checklist for completion.

**Additional file 2.** Table A. Representative Quotes from Themes Within each Implementation Support, Years 1 and 2.

## Data Availability

The datasets generated and/or analyzed during the current study are not publicly available due to security provisions of the protocol approved by the institutional review board, but de-identified data may be available from the corresponding author on reasonable request.

## Abbreviations

PBIS: Positive Behavioral Interventions and Supports
EBIs: Evidence-based interventions
RS3: Rural School Support Strategies
QIF: Quality Implementation Framework
ISP: Implementation support practitioner
TA: Technical assistance
