# Supplementary material for: Acceptability, appropriateness, and feasibility of Rural School Support Strategies for behavioral interventions: a mixed methods evaluation over two years of a hybrid type 3 implementation-effectiveness trial
Source: Implement Sci Commun. 2023 Aug 11;4:92. doi: 10.1186/s43058-023-00478-4 (PMC10416374; doi:10.1186/s43058-023-00478-4)
Supplement: Supplementary file 2 — Additional file 2. Table A. Representative Quotes from Themes Within each Implementation Support, Years 1 and 2. [file 43058_2023_478_MOESM2_ESM.docx]

**Table A. Representative Quotes from Themes Within each Implementation Support, Years 1 and 2**

| **Theme(s)** | **Representative Quote(s)** |
| --- | --- |
| **Technical Assistance from the Implementation Support Specialist (ISS)** | |
| **Knowledge:** Participants said advice the ISS gave was helpful and their expertise was valued. Many mentioned that the ISS always knew where to point the team for resources on a particular subject. | **Year 1:**  "And he has often been able to come up with some very insightful perspective to help us change the way we're thinking, to go back to relationship development. When you’re kind of… in the weeds, if you will… with a student or two, it can become very draining and you do lose that open-minded ‘we love all these kids’ feeling. So, I personally think without that resource, it would not have gone as well as it did. People would get out into the weeds and stay there."  “He did remind us a lot about the different videos and things that we could watch online. So that was pretty helpful.” |
| **Presence:** Knowing the ISS was always there to answer questions if needed was helpful for teams. Knowing the ISS would attend (physically or virtually) the team meetings spurred teams to get their agendas and data in order, and come ready with questions. | **Year 1:**  "And then overall, [the ISS] was just always available and willing and ready to help even when there are things that we need to improve on, it's always positive. I wish I had that talent, to be able to help direct and guide and maybe recorrect in such a nice way. But I've appreciated him reaching out to me and just saying, ‘you're doing a great job’ or ‘how are things going?’ I've just always felt supported by it."  "Having the constant meetings with [the ISS] was super helpful and he was never in an ‘I got you’ state, but in a mentorship and guiding, and a third party looking above and seeing what he sees objectively which was super nice."  **Year 2:**  “I think it's great to have him be virtual, too bad he's not here in person, but it's been nice to have his support”  “I think it helps keep us on track and helps us check up on how we're progressing with looking at our data, seeing what issues there are, seeing if there's any kids that need any further intervention. It's just been, it's been great to have that. It's just it's a great support.”  “It's given me a springboard to say, 'Hey, you know, we need to have time for this meeting. We need to have time for this data, we need to have time.'”  “I don't think we could have done it without Nate. [Laughter] He's been such a big help. He answers all our questions. And he's helped me a lot with the data side of things. I've had a lot of questions about SWIS. My counselor and I both, we're both learning, her the tier two side, me the tier one side. Nate has been there for all our questions and and we've had some behaviors come up this year that we don't really know how to deal with and Nate's really helped us brainstorm, you know, some positive things we could do to deal with those behaviors that we need to fix. So it's been really crucial. His role has been really critical to our success.” |
| **Relationship:** Personal relationship with members of the PBIS team, particularly coaches, built trust between the ISS and schools. Additionally, the juxtaposition of the ISS as external to the school gave them additional clout, according to coaches and administrators. | **Year 1:**  “It was nice having them be present. I know it's a long drive and we're out in the sticks, but it was nice. I felt like the teachers kind of got to know him. Like, the other teachers who didn't go to the trainings kind of got to know him because he would come at our lunch and sit down. I think they felt very comfortable asking him questions. So that was kind of nice. I think it kind of built a little bit of a relationship. You know, now that it's kind of already there, I think that they would feel comfortable to continue to ask him questions virtually. But I think at the beginning it was really nice to have him there.”  “He's a very positive person, which is fabulous. I mean, I admire him.”  “He was great at bringing a ton of guidance especially early on when we weren’t really knowing what we were doing.”  “I thought it was really critical. Like when he presents to staff, we did have him attend a staff meeting too, not just the first part of the year, I think he might have come to one or two of those. Teachers tend to really respect outside resources. The outside expert tends to carry more weight with teachers and it's nice to have him here because they listen to what he has to say. He gains respect fairly easily. He’s been really good in guiding.”  **Year 2:**  “It’s just really awesome to have somebody who has kind of a bigger picture and they get to talk and see how this is working in other schools. We don’t have that. And so he will give us ideas for our choices, our menu. He’ll give us just examples of how other schools are handling this same type of situation. And oftentimes, we’ve made something out of nothing that he’s like, ‘Oh, that’s not a big deal. Just do this and we’ll fix it'. But he, they, they’re great. Like, they’ve always been super good to respond, help us out. I just don’t know what we would have done without them. It wouldn’t be the same.” |
| **Communication Style:** Many coaches and administrators stated that they valued the way the ISS interacted with their teams. Comments were always delivered positively, modeling what the ISS was teaching the team. | **Year 1:**  "He's very skilled at coaching. Asking those questions in a constructivist way so that teachers are thinking deeply about their practice and not just giving advice. Like making sure they're thinking about what's working and what's not working."  "So, from conversations, to his emails of follow-up, to, you know, just being non-directive and supportive and very well thought out, comments of how to support us as a staff, it was not only preached but practiced." |
| **Delivery Method:** In terms of delivery, face to face was widely preferred over virtual, or interacting over email or phone. In-person visits allowed for relationship-building not just with the PBIS team, but with the whole school staff. | **Year 1:**  “The in-person visits really helped us feel connected to the movement."  "He would be able to observe and give feedback. Like I think he single-handedly transformed our PE teacher’s perspective on classroom management and all of those conversations are driven by observations, right? So, if you can't be in a classroom to really see what's on the walls and see how teachers are kind of using those practices it's pretty tricky to the depth of, I guess, pushing us forward. I felt like when he was on-site, we were moving at a faster pace of implementation versus when he was off site."  “It’s just like you and I, if we were to sit in the room right now, we would connect a little differently than this. So maybe a little differently. But I think he had established relationships with us enough beforehand, before he went to the online, that it wasn't major. Face to face is more powerful.” |
| **Flexibility:** Taking a gentler approach toward aiding schools in implementation, and supporting whatever need that arose for schools. | **Year 2:**  “We've had him involved in, I think maybe only one, might be two, but I think really one Zoom meeting, when we were having a team meeting… And, and so, yeah, I think they've done well we just haven't involved them as much as we should probably.” |
| **Virtual Learning Sessions** | |
| **Review:** The opportunity to have more time to digest particular topics, and suggest topics that they wanted covered, was valued by coaches. In year 2 the focus on self-care concepts was valued. | **Year 1:**  “The virtual trainings gave us another chance to learn it at a different time than the first round of all these things. So, I think it's valuable.”  **Year 2:**  “I like that they kind of refresh my brain and every month remind me to not put PBIS at the back of my mind, but kind of give us those refreshers, especially some of the things they're talking about from the, you know, from the summer trainings that we did. So that's been really nice. Yeah, I feel like they just keep us rolling.”  "'We're learning about just taking care of ourselves for PBIS!' Like, I my mind was really quite blown and I was really impressed that that would be our focus, when we're trying to implement and roll out the next tier and keep things going. And so I really appreciated that focus. And it helped me as a coach to then know how to go and better help my team.”  “The biggest one that that had an impact on it was just taking care of, your self-care. That, after I received that training, the next day, I just sent that out to the, to the PBIS team. I said, this seems like a really, it came at a really timely spot in, right at the, I want to say that was about October. And there's just, tensions were high. And after I talked with them, we decided, okay, next faculty meeting, we're gonna bring this up, everybody got one of those self-care sheets? And it just, yeah, absolutely, so they've really helped.” |
| **Collaboration:** Coaches expressed that it was helpful to troubleshoot PBIS implementation problems with coaches from other schools and bounce ideas off each other. Collaboration was even more valued in year 2. | **Year 1:**  “Being able to chat with other people, even throughout the state or even within my district was super helpful, because it was nice to know that we weren't alone if we had an issue. And it was also nice to just share ideas, and it just helps to keep your ideas fresh, I guess. So, I really think that that part is important.”  “I thought that the coaching sessions were good. When we were doing all of the virtual learning that way I thought that was good, where we could definitely interact with other coaches and see what they were dealing with in their schools, how different from what we were dealing with.”  “So, we had a unique experience that other people wanted to know about. And we wanted to know about what it looks like in their school. So those are the things, anytime those kinds of conversations could happen. I think those are the biggest resources.”  **Year 2:**  “I've loved the monthly meetings as well, just to hear what other schools are doing and have a chance to reflect a little bit and talk to other PBIS coaches and not feel so alone in it and isolated.”  “I like them. I'm starting to know different people better. And I, there are certain ones that I identified more with because I, there are more similar to what we are. But I like to be able to talk to different people about different ideas and how things may be working for them that aren't working for us, or vice versa.” |
| **Format:** The digital interface of the virtual learning sessions was typically a drawback, either because online learning made it harder to block out distractions, or it was not their preference. The remote sessions were better received in year 2. | **Year 1:**  “I'm not a fan of the webinars, I missed several of them because every time I would log on I would get interrupted. I guess for me, give me the resource, I'll read it, or and I'll or I'll watch it and then I'll ask questions. But just to listen to someone, facilitate for an hour when it's something that I could read on my own, I'd prefer the other.”  “The only thing that I struggle with a lot, just personally, I'm a hands-on learner. I'm not an online learner. So, like the Check-In/Check-Out [a PBIS component] thing kind of stressed me out. Because I like people to be next to me, like, ‘is this how you do it?’ And I know you can interact, but I was just so overwhelmed that I didn't say anything. You know what I mean? I was kind of just petrified. But, and then of course, I emailed [the ISS] afterwards.  **Year 2**:  “Sometimes there's an obstacle, but I always have my phone on me. So I can always listen in the car. There have been some of those virtual sessions where I am on the move, they're been, they've been a lot different this year, too. So I can kind of be more flexible with being on the move and listening in the car.”  “I am not a fan of the virtual meetings. In fact, half the time I forget to get on them, like I plan to go and then I get busy. You know, especially not having a prep, like after school, I'm making copies, I'm doing the stuff I need to do… I don't care for the format anyway. I mean, not that it's all about me, but I just don't learn well that way… If you have to [have] it virtual just send me the presentation and the notes and let me read it and I'm good.” |
| **Web Portal** | |
| **Review:** The portal was cited as a helpful resource repository, when participants thought to use it. | **Year 1:**  “I feel like the area where we could improve was using the website. We used that kind of sparingly and I think that we had a lot of things going on and adding that I think in the middle of the year they had it all where you could go onto the site, do all that and we looked at it, but we didn't utilize that probably to its fullest.”  “I loved when they put up the, I think they call it the RK-12 portal and we could access all those materials that they were sharing with us. That was huge, because there were some videos and some documents and things that we really wanted to share with staff and instead of like, emailing [the ISS] to get them to us, we could just hop on there and find everything we needed. That was huge. I don't know from the coach's perspective on the rest of the stuff accessed or used regularly, but I know for the team that was really helpful.”  “We did use the website quite a bit, the website resources, especially the rest of our team for different note taking things, flowcharts examples, all that kind of stuff. We looked at that quite a bit.” |
| **Other** | |
| **Team Work Time:**  PBIS teams having the opportunity to sit down with each other with allocated time and tasks allowed them to work on planning and materials for the upcoming school year. | **Year 1:**  “When we had the summer workshop series, those are great when you can have your whole team there… and that's where a lot of the heavy lifting came in. I think that really clarified for the entire team what the mission was and what that roadmap looked like and so again we came in unified, you know, if anyone on the team was like ‘well, yeah, I don't think that's really gonna work.’ You know, everyone felt really positive.” |
| **Quality:** Participants said that there was a lot of high-quality content at the training, and teams liked attending the training. However, the amount of information also made it difficult to digest for some. | **Year 1:**  “I did go through a few webinars to remind myself. We were trained obviously on the SWIS, but then when it came down to do it again, it was like, oh, I forgot everything. And I was really lucky to have those webinars.”  “I watched some of the video tutorials and those kinds of things to help refresh my memory because we did the SWIS training in the summer and then even in the spring it was months before I really actually got to put it in place to start using it. So those were nice to have available.” |
